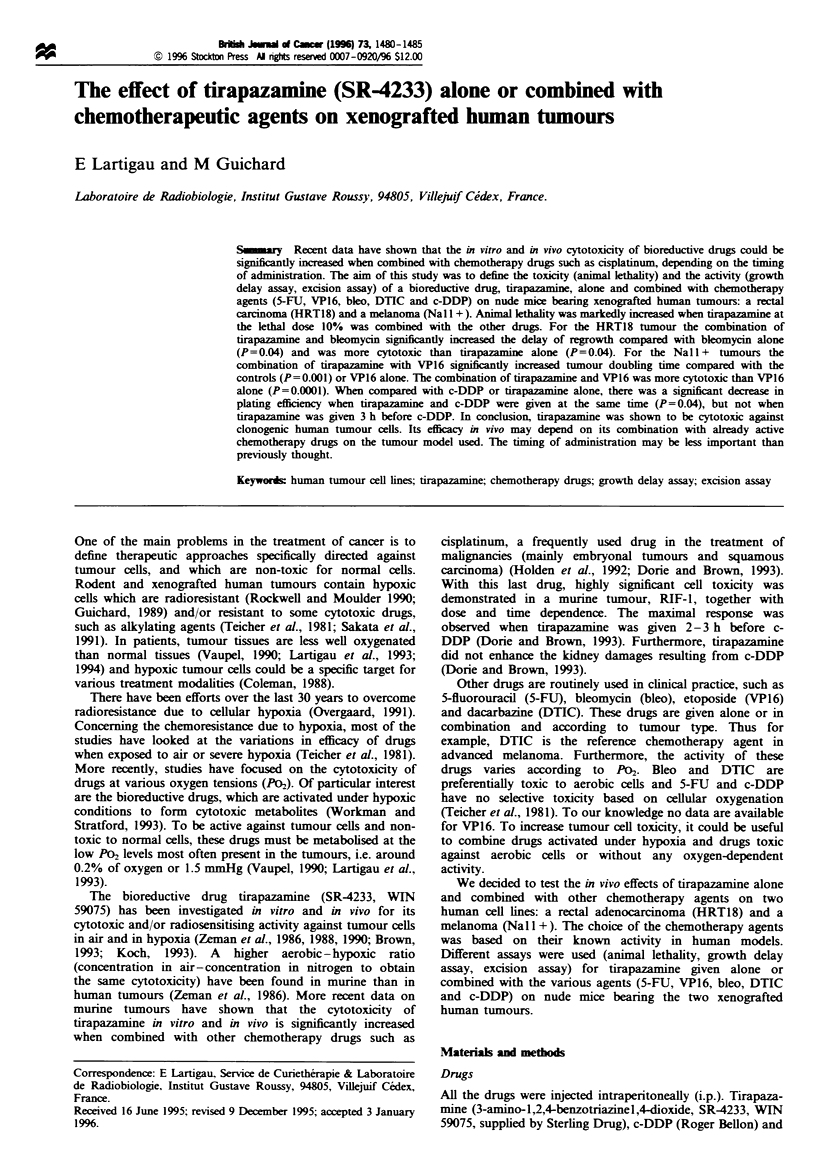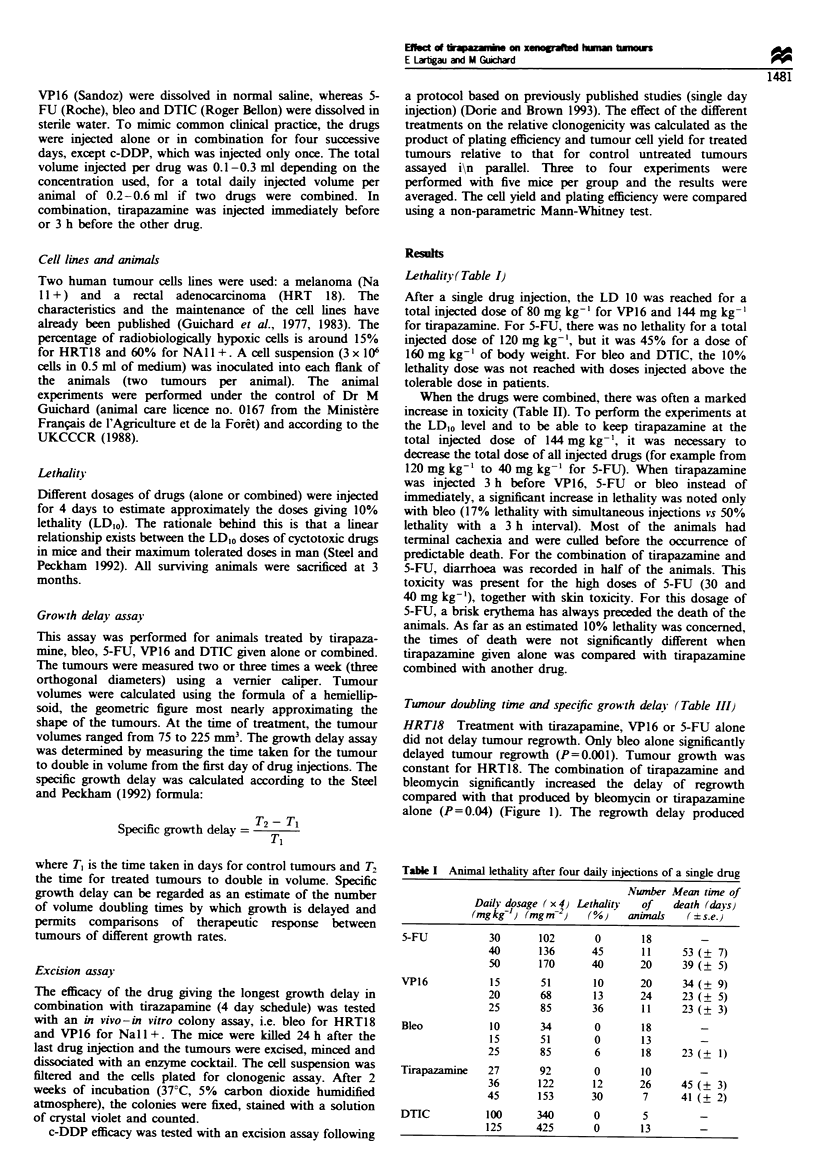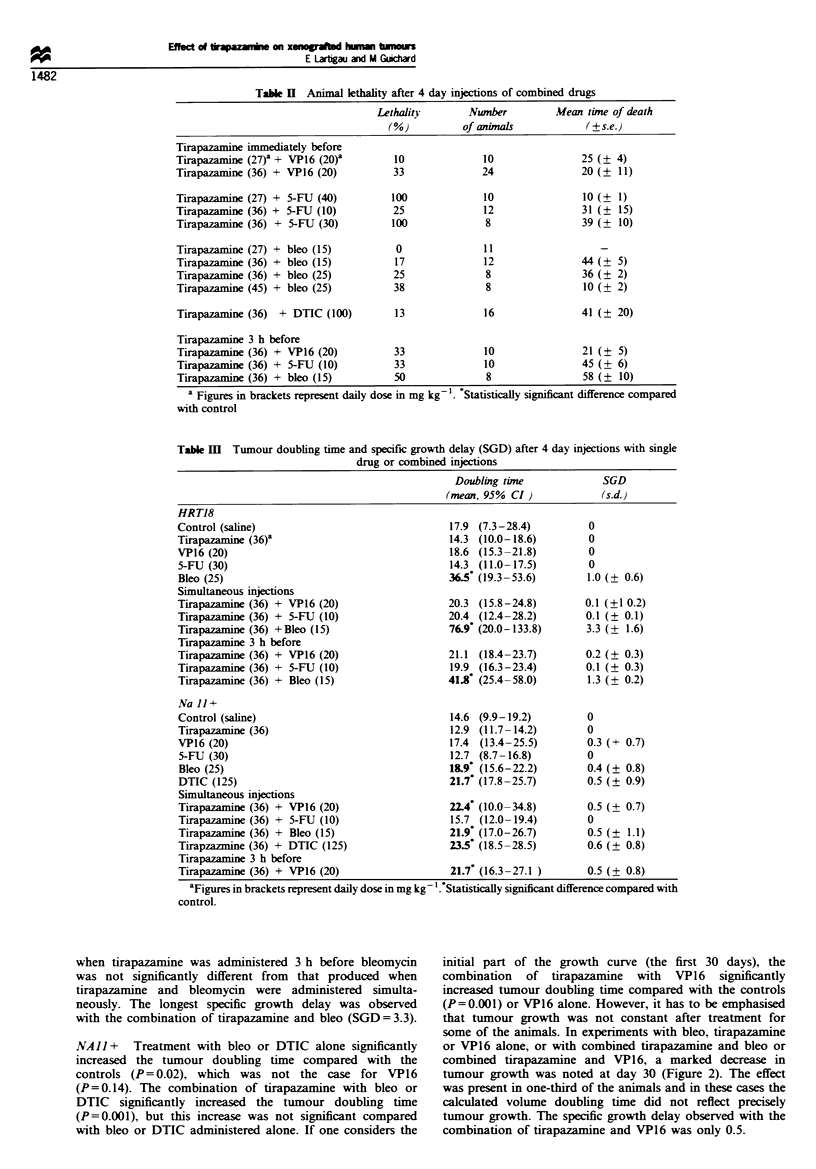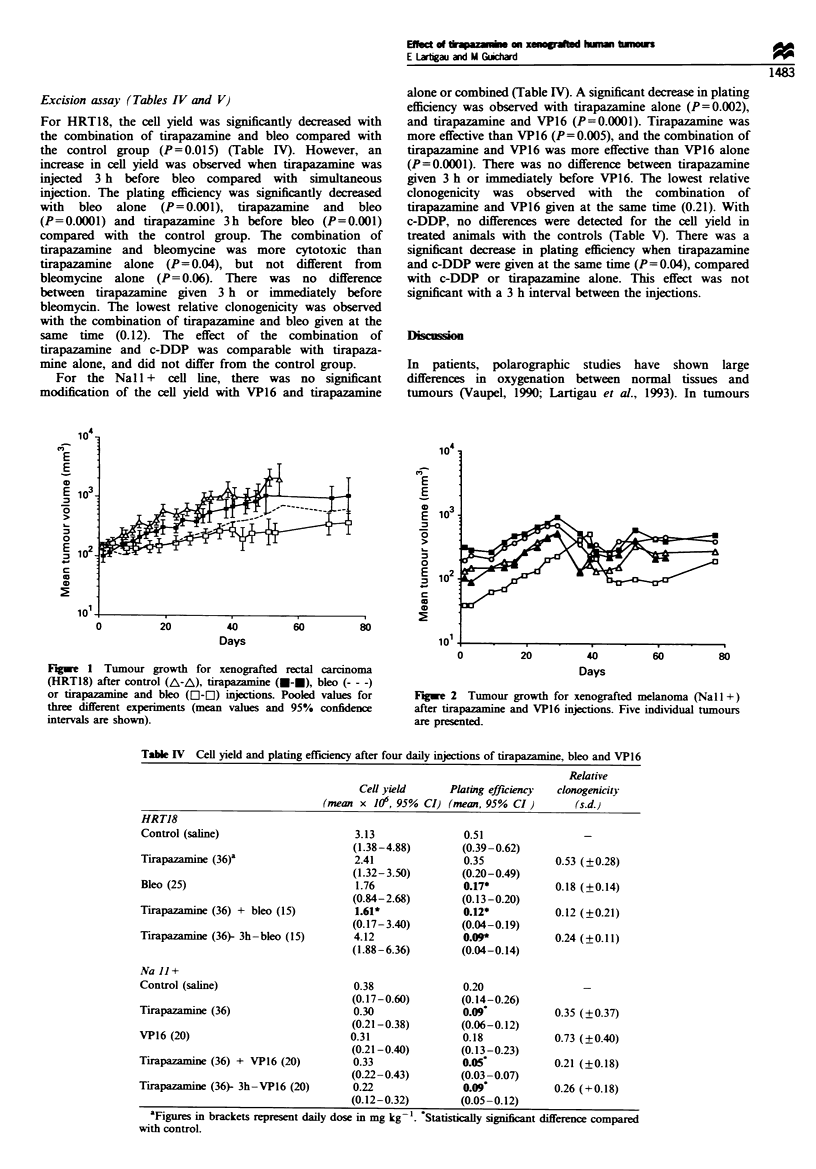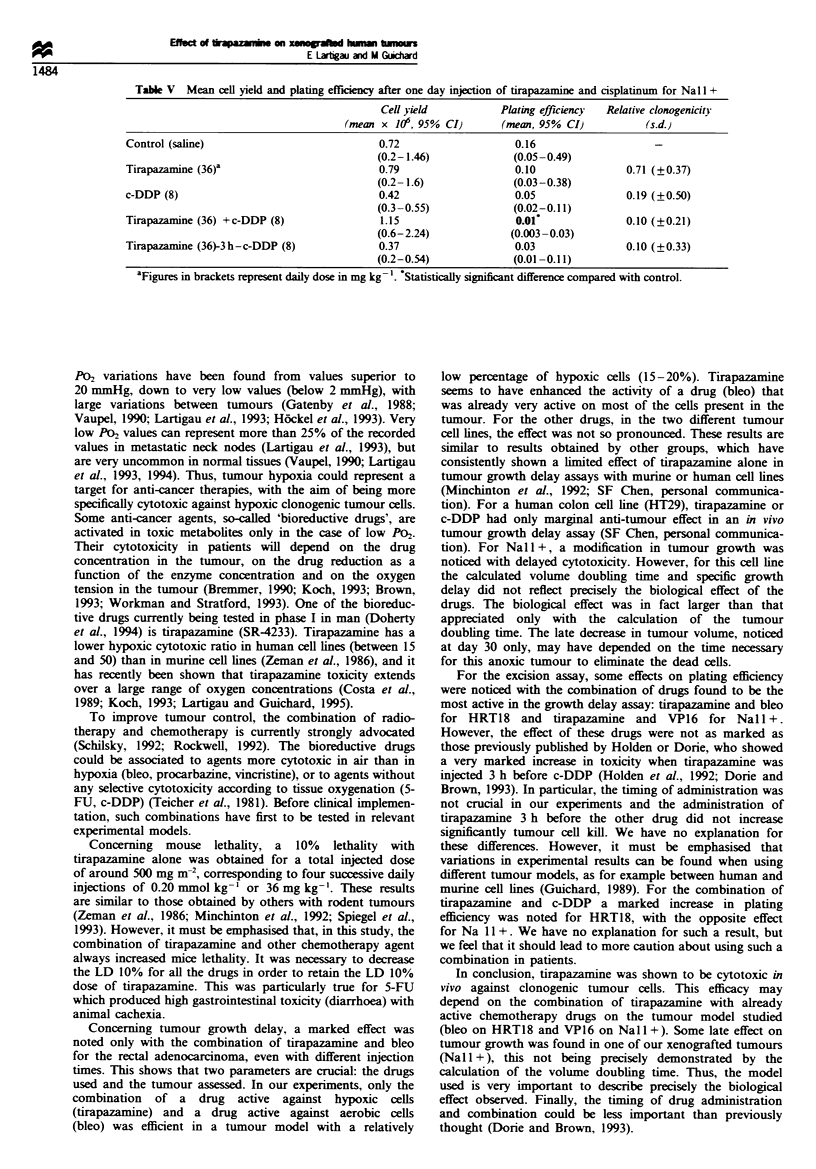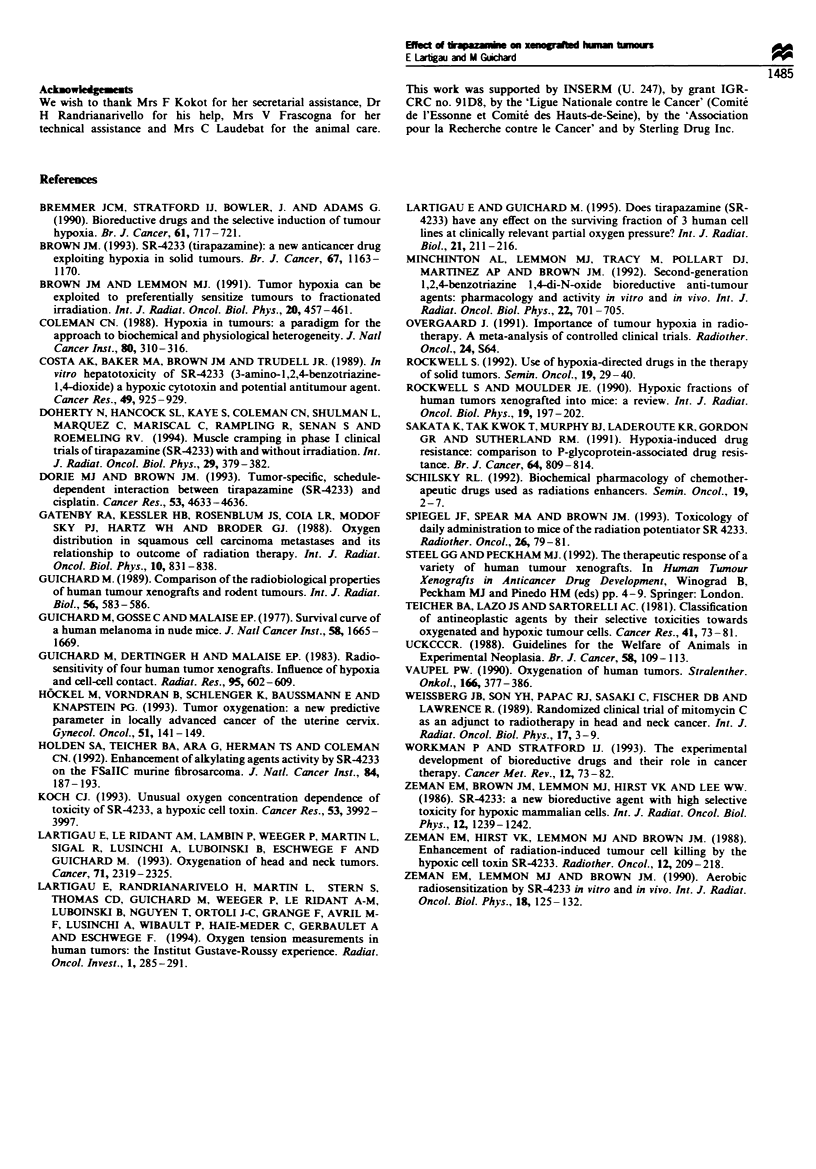# The effect of tirapazamine (SR-4233) alone or combined with chemotherapeutic agents on xenografted human tumours.

**DOI:** 10.1038/bjc.1996.280

**Published:** 1996-06

**Authors:** E. Lartigau, M. Guichard

**Affiliations:** Laboratoire de Radiobiologie, Institut Gustave Roussy, Villejuif, France.

## Abstract

Recent data have shown that the in vitro and in vivo cytotoxicity of bioreductive drugs could be significantly increased when combined with chemotherapy drugs such as cisplatinum, depending on the timing of administration. The aim of this study was to define the toxicity (animal lethality) and the activity (growth delay assay, excision assay) of a bioreductive drug, tirapazamine, alone and combined with chemotherapy agents (5-FU, VP16, bleo, DTIC and c-DDP) on nude mice bearing xenografted human tumours: a rectal carcinoma (HRT18) and a melanoma (Na11+). Animal lethality was markedly increased when tirapazamine at the lethal dose 10% was combined with the other drugs. For the HRT18 tumour the combination of tirapazamine and bleomycin significantly increased the delay of regrowth compared with bleomycin alone (P = 0.04) and was more cytotoxic than tirapazamine alone (P = 0.04). For the Na11+ tumours the combination of tirapazamine with VP16 significantly increased tumour doubling time compared with the controls (P = 0.001) or VP16 alone. The combination of tirapazamine and VP16 was more cytotoxic than VP16 alone (P = 0.0001). When compared with c-DDP or tirapazamine alone, there was a significant decrease in plating efficiency when tirapazamine and c-DDP were given at the same time (P = 0.04), but not when tirapazamine was given 3 h before c-DDP. In conclusion, tirapazamine was shown to be cytotoxic against clonogenic human tumour cells. Its efficacy in vivo may depend on its combination with already active chemotherapy drugs on the tumour model used. The timing of administration may be less important than previously thought.


© 1996 Stockton Press All rights reserved 0007-0920/96 $12.00

### The effect of tirapazamine (SR-4233) alone or combined with chemotherapeutic agents on xenografted human tumours

E Lartigau and M Guichard

*Laboratoire de Radiobiologie, Institut Gustave Roussy, 94805, Villejuif Cédex, France.*

**Summary** Recent data have shown that the *in vitro* and *in vivo* cytotoxicity of bioreductive drugs could be significantly increased when combined with chemotherapy drugs such as cisplatinum, depending on the timing of administration. The aim of this study was to define the toxicity (animal lethality) and the activity (growth delay assay, excision assay) of a bioreductive drug, tirapazamine, alone and combined with chemotherapy agents (5-FU, VP16, bleo, DTIC and c-DDP) on nude mice bearing xenografted human tumours: a rectal carcinoma (HRT18) and a melanoma (Na11+). Animal lethality was markedly increased when tirapazamine at the lethal dose 10% was combined with the other drugs. For the HRT18 tumour the combination of tirapazamine and bleomycin significantly increased the delay of regrowth compared with bleomycin alone ($P=0.04$) and was more cytotoxic than tirapazamine alone ($P=0.04$). For the Na11+ tumours the combination of tirapazamine with VP16 significantly increased tumour doubling time compared with the controls ($P=0.001$) or VP16 alone. The combination of tirapazamine and VP16 was more cytotoxic than VP16 alone ($P=0.0001$). When compared with c-DDP or tirapazamine alone, there was a significant decrease in plating efficiency when tirapazamine and c-DDP were given at the same time ($P=0.04$), but not when tirapazamine was given 3 h before c-DDP. In conclusion, tirapazamine was shown to be cytotoxic against clonogenic human tumour cells. Its efficacy *in vivo* may depend on its combination with already active chemotherapy drugs on the tumour model used. The timing of administration may be less important than previously thought.

**Keywords:** human tumour cell lines; tirapazamine; chemotherapy drugs; growth delay assay; excision assay

One of the main problems in the treatment of cancer is to define therapeutic approaches specifically directed against tumour cells, and which are non-toxic for normal cells. Rodent and xenografted human tumours contain hypoxic cells which are radioresistant (Rockwell and Moulder 1990; Guichard, 1989) and/or resistant to some cytotoxic drugs, such as alkylating agents (Teicher *et al.*, 1981; Sakata *et al.*, 1991). In patients, tumour tissues are less well oxygenated than normal tissues (Vaupel, 1990; Lartigau *et al.*, 1993; 1994) and hypoxic tumour cells could be a specific target for various treatment modalities (Coleman, 1988).

There have been efforts over the last 30 years to overcome radioresistance due to cellular hypoxia (Overgaard, 1991). Concerning the chemoresistance due to hypoxia, most of the studies have looked at the variations in efficacy of drugs when exposed to air or severe hypoxia (Teicher *et al.*, 1981). More recently, studies have focused on the cytotoxicity of drugs at various oxygen tensions ($Po_2$). Of particular interest are the bioreductive drugs, which are activated under hypoxic conditions to form cytotoxic metabolites (Workman and Stratford, 1993). To be active against tumour cells and non-toxic to normal cells, these drugs must be metabolised at the low $Po_2$ levels most often present in the tumours, i.e. around 0.2% of oxygen or 1.5 mmHg (Vaupel, 1990; Lartigau *et al.*, 1993).

The bioreductive drug tirapazamine (SR-4233, WIN 59075) has been investigated *in vitro* and *in vivo* for its cytotoxic and/or radiosensitising activity against tumour cells in air and in hypoxia (Zeman *et al.*, 1986, 1988, 1990; Brown, 1993; Koch, 1993). A higher aerobic–hypoxic ratio (concentration in air–concentration in nitrogen to obtain the same cytotoxicity) have been found in murine than in human tumours (Zeman *et al.*, 1986). More recent data on murine tumours have shown that the cytotoxicity of tirapazamine *in vitro* and *in vivo* is significantly increased when combined with other chemotherapy drugs such as cisplatinum, a frequently used drug in the treatment of malignancies (mainly embryonal tumours and squamous carcinoma) (Holden *et al.*, 1992; Dorie and Brown, 1993). With this last drug, highly significant cell toxicity was demonstrated in a murine tumour, RIF-1, together with dose and time dependence. The maximal response was observed when tirapazamine was given 2–3 h before c-DDP (Dorie and Brown, 1993). Furthermore, tirapazamine did not enhance the kidney damages resulting from c-DDP (Dorie and Brown, 1993).

Other drugs are routinely used in clinical practice, such as 5-fluorouracil (5-FU), bleomycin (bleo), etoposide (VP16) and dacarbazine (DTIC). These drugs are given alone or in combination and according to tumour type. Thus for example, DTIC is the reference chemotherapy agent in advanced melanoma. Furthermore, the activity of these drugs varies according to $Po_2$. Bleo and DTIC are preferentially toxic to aerobic cells and 5-FU and c-DDP have no selective toxicity based on cellular oxygenation (Teicher *et al.*, 1981). To our knowledge no data are available for VP16. To increase tumour cell toxicity, it could be useful to combine drugs activated under hypoxia and drugs toxic against aerobic cells or without any oxygen-dependent activity.

We decided to test the *in vivo* effects of tirapazamine alone and combined with other chemotherapy agents on two human cell lines: a rectal adenocarcinoma (HRT18) and a melanoma (Na11+). The choice of the chemotherapy agents was based on their known activity in human models. Different assays were used (animal lethality, growth delay assay, excision assay) for tirapazamine given alone or combined with the various agents (5-FU, VP16, bleo, DTIC and c-DDP) on nude mice bearing the two xenografted human tumours.

Correspondence: E Lartigau, Service de Curiethérapie & Laboratoire de Radiobiologie, Institut Gustave Roussy, 94805, Villejuif Cédex, France.
Received 16 June 1995; revised 9 December 1995; accepted 3 January 1996.

### Materials and methods

### *Drugs*

All the drugs were injected intraperitoneally (i.p.). Tirapazamine (3-amino-1,2,4-benzotriazine1,4-dioxide, SR-4233, WIN 59075, supplied by Sterling Drug), c-DDP (Roger Bellon) and

VP16 (Sandoz) were dissolved in normal saline, whereas 5-FU (Roche), bleo and DTIC (Roger Bellon) were dissolved in sterile water. To mimic common clinical practice, the drugs were injected alone or in combination for four successive days, except c-DDP, which was injected only once. The total volume injected per drug was 0.1–0.3 ml depending on the concentration used, for a total daily injected volume per animal of 0.2–0.6 ml if two drugs were combined. In combination, tirapazamine was injected immediately before or 3 h before the other drug.

*Cell lines and animals*

Two human tumour cells lines were used: a melanoma (Na 11+) and a rectal adenocarcinoma (HRT 18). The characteristics and the maintenance of the cell lines have already been published (Guichard *et al.*, 1977, 1983). The percentage of radiobiologically hypoxic cells is around 15% for HRT18 and 60% for NA11+. A cell suspension ($3 \times 10^6$ cells in 0.5 ml of medium) was inoculated into each flank of the animals (two tumours per animal). The animal experiments were performed under the control of Dr M Guichard (animal care licence no. 0167 from the Ministère Français de l'Agriculture et de la Forêt) and according to the UKCCCR (1988).

*Lethality*

Different dosages of drugs (alone or combined) were injected for 4 days to estimate approximately the doses giving 10% lethality ($LD_{10}$). The rationale behind this is that a linear relationship exists between the $LD_{10}$ doses of cyctotoxic drugs in mice and their maximum tolerated doses in man (Steel and Peckham 1992). All surviving animals were sacrificed at 3 months.

*Growth delay assay*

This assay was performed for animals treated by tirapazamine, bleo, 5-FU, VP16 and DTIC given alone or combined. The tumours were measured two or three times a week (three orthogonal diameters) using a vernier caliper. Tumour volumes were calculated using the formula of a hemiellipsoid, the geometric figure most nearly approximating the shape of the tumours. At the time of treatment, the tumour volumes ranged from 75 to 225 mm$^3$. The growth delay assay was determined by measuring the time taken for the tumour to double in volume from the first day of drug injections. The specific growth delay was calculated according to the Steel and Peckham (1992) formula:

$$\text{Specific growth delay} = \frac{T_2 - T_1}{T_1}$$

where $T_1$ is the time taken in days for control tumours and $T_2$ the time for treated tumours to double in volume. Specific growth delay can be regarded as an estimate of the number of volume doubling times by which growth is delayed and permits comparisons of therapeutic response between tumours of different growth rates.

*Excision assay*

The efficacy of the drug giving the longest growth delay in combination with tirazapamine (4 day schedule) was tested with an *in vivo–in vitro* colony assay, i.e. bleo for HRT18 and VP16 for Na11+. The mice were killed 24 h after the last drug injection and the tumours were excised, minced and dissociated with an enzyme cocktail. The cell suspension was filtered and the cells plated for clonogenic assay. After 2 weeks of incubation (37°C, 5% carbon dioxide humidified atmosphere), the colonies were fixed, stained with a solution of crystal violet and counted.

c-DDP efficacy was tested with an excision assay following a protocol based on previously published studies (single day injection) (Dorie and Brown 1993). The effect of the different treatments on the relative clonogenicity was calculated as the product of plating efficiency and tumour cell yield for treated tumours relative to that for control untreated tumours assayed i\n parallel. Three to four experiments were performed with five mice per group and the results were averaged. The cell yield and plating efficiency were compared using a non-parametric Mann-Whitney test.

### Results

*Lethality (Table I)*

After a single drug injection, the LD 10 was reached for a total injected dose of 80 mg kg$^{-1}$ for VP16 and 144 mg kg$^{-1}$ for tirapazamine. For 5-FU, there was no lethality for a total injected dose of 120 mg kg$^{-1}$, but it was 45% for a dose of 160 mg kg$^{-1}$ of body weight. For bleo and DTIC, the 10% lethality dose was not reached with doses injected above the tolerable dose in patients.

When the drugs were combined, there was often a marked increase in toxicity (Table II). To perform the experiments at the $LD_{10}$ level and to be able to keep tirapazamine at the total injected dose of 144 mg kg$^{-1}$, it was necessary to decrease the total dose of all injected drugs (for example from 120 mg kg$^{-1}$ to 40 mg kg$^{-1}$ for 5-FU). When tirapazamine was injected 3 h before VP16, 5-FU or bleo instead of immediately, a significant increase in lethality was noted only with bleo (17% lethality with simultaneous injections *vs* 50% lethality with a 3 h interval). Most of the animals had terminal cachexia and were culled before the occurrence of predictable death. For the combination of tirapazamine and 5-FU, diarrhoea was recorded in half of the animals. This toxicity was present for the high doses of 5-FU (30 and 40 mg kg$^{-1}$), together with skin toxicity. For this dosage of 5-FU, a brisk erythema has always preceded the death of the animals. As far as an estimated 10% lethality was concerned, the times of death were not significantly different when tirapazamine given alone was compared with tirapazamine combined with another drug.

*Tumour doubling time and specific growth delay (Table III)*

*HRT18* Treatment with tirazapamine, VP16 or 5-FU alone did not delay tumour regrowth. Only bleo alone significantly delayed tumour regrowth ($P = 0.001$). Tumour growth was constant for HRT18. The combination of tirapazamine and bleomycin significantly increased the delay of regrowth compared with that produced by bleomycin or tirapazamine alone ($P = 0.04$) (Figure 1). The regrowth delay produced

**Table I** Animal lethality after four daily injections of a single drug

| | Daily dosage (×4) (mg kg$^{-1}$) | (mg m$^{-2}$) | Lethality (%) | Number of animals | Mean time of death (days) (±s.e.) |
|---|---|---|---|---|---|
| 5-FU | 30 | 102 | 0 | 18 | – |
| | 40 | 136 | 45 | 11 | 53 (± 7) |
| | 50 | 170 | 40 | 20 | 39 (± 5) |
| VP16 | 15 | 51 | 10 | 20 | 34 (± 9) |
| | 20 | 68 | 13 | 24 | 23 (± 5) |
| | 25 | 85 | 36 | 11 | 23 (± 3) |
| Bleo | 10 | 34 | 0 | 18 | – |
| | 15 | 51 | 0 | 13 | – |
| | 25 | 85 | 6 | 18 | 23 (± 1) |
| Tirapazamine | 27 | 92 | 0 | 10 | – |
| | 36 | 122 | 12 | 26 | 45 (± 3) |
| | 45 | 153 | 30 | 7 | 41 (± 2) |
| DTIC | 100 | 340 | 0 | 5 | – |
| | 125 | 425 | 0 | 13 | – |

Table II Animal lethality after 4 day injections of combined drugs

| | Lethality (%) | Number of animals | Mean time of death (±s.e.) |
|---|---|---|---|
| Tirapazamine immediately before | | | |
| Tirapazamine (27)[a] + VP16 (20)[a] | 10 | 10 | 25 (± 4) |
| Tirapazamine (36) + VP16 (20) | 33 | 24 | 20 (± 11) |
| Tirapazamine (27) + 5-FU (40) | 100 | 10 | 10 (± 1) |
| Tirapazamine (36) + 5-FU (10) | 25 | 12 | 31 (± 15) |
| Tirapazamine (36) + 5-FU (30) | 100 | 8 | 39 (± 10) |
| Tirapazamine (27) + bleo (15) | 0 | 11 | – |
| Tirapazamine (36) + bleo (15) | 17 | 12 | 44 (± 5) |
| Tirapazamine (36) + bleo (25) | 25 | 8 | 36 (± 2) |
| Tirapazamine (45) + bleo (25) | 38 | 8 | 10 (± 2) |
| Tirapazamine (36) + DTIC (100) | 13 | 16 | 41 (± 20) |
| Tirapazamine 3 h before | | | |
| Tirapazamine (36) + VP16 (20) | 33 | 10 | 21 (± 5) |
| Tirapazamine (36) + 5-FU (10) | 33 | 10 | 45 (± 6) |
| Tirapazamine (36) + bleo (15) | 50 | 8 | 58 (± 10) |

[a] Figures in brackets represent daily dose in mg $kg^{-1}$. *Statistically significant difference compared with control

Table III Tumour doubling time and specific growth delay (SGD) after 4 day injections with single drug or combined injections

| | Doubling time (mean, 95% CI) | SGD (s.d.) |
|---|---|---|
| *HRT18* | | |
| Control (saline) | 17.9 (7.3–28.4) | 0 |
| Tirapazamine (36)[a] | 14.3 (10.0–18.6) | 0 |
| VP16 (20) | 18.6 (15.3–21.8) | 0 |
| 5-FU (30) | 14.3 (11.0–17.5) | 0 |
| Bleo (25) | **36.5*** (19.3–53.6) | 1.0 (± 0.6) |
| Simultaneous injections | | |
| Tirapazamine (36) + VP16 (20) | 20.3 (15.8–24.8) | 0.1 (±1 0.2) |
| Tirapazamine (36) + 5-FU (10) | 20.4 (12.4–28.2) | 0.1 (± 0.1) |
| Tirapazamine (36) + Bleo (15) | **76.9*** (20.0–133.8) | 3.3 (± 1.6) |
| Tirapazamine 3 h before | | |
| Tirapazamine (36) + VP16 (20) | 21.1 (18.4–23.7) | 0.2 (± 0.3) |
| Tirapazamine (36) + 5-FU (10) | 19.9 (16.3–23.4) | 0.1 (± 0.3) |
| Tirapazamine (36) + Bleo (15) | **41.8*** (25.4–58.0) | 1.3 (± 0.2) |
| *Na 11+* | | |
| Control (saline) | 14.6 (9.9–19.2) | 0 |
| Tirapazamine (36) | 12.9 (11.7–14.2) | 0 |
| VP16 (20) | 17.4 (13.4–25.5) | 0.3 (+ 0.7) |
| 5-FU (30) | 12.7 (8.7–16.8) | 0 |
| Bleo (25) | **18.9*** (15.6–22.2) | 0.4 (± 0.8) |
| DTIC (125) | **21.7*** (17.8–25.7) | 0.5 (± 0.9) |
| Simultaneous injections | | |
| Tirapazamine (36) + VP16 (20) | **22.4*** (10.0–34.8) | 0.5 (± 0.7) |
| Tirapazamine (36) + 5-FU (10) | 15.7 (12.0–19.4) | 0 |
| Tirapazamine (36) + Bleo (15) | **21.9*** (17.0–26.7) | 0.5 (± 1.1) |
| Tirapzazmine (36) + DTIC (125) | **23.5*** (18.5–28.5) | 0.6 (± 0.8) |
| Tirapazamine 3 h before | | |
| Tirapazamine (36) + VP16 (20) | **21.7*** (16.3–27.1) | 0.5 (± 0.8) |

[a]Figures in brackets represent daily dose in mg $kg^{-1}$. *Statistically significant difference compared with control.

when tirapazamine was administered 3 h before bleomycin was not significantly different from that produced when tirapazamine and bleomycin were administered simultaneously. The longest specific growth delay was observed with the combination of tirapazamine and bleo (SGD = 3.3).

*NA11+* Treatment with bleo or DTIC alone significantly increased the tumour doubling time compared with the controls ($P = 0.02$), which was not the case for VP16 ($P = 0.14$). The combination of tirapazamine with bleo or DTIC significantly increased the tumour doubling time ($P = 0.001$), but this increase was not significant compared with bleo or DTIC administered alone. If one considers the initial part of the growth curve (the first 30 days), the combination of tirapazamine with VP16 significantly increased tumour doubling time compared with the controls ($P = 0.001$) or VP16 alone. However, it has to be emphasised that tumour growth was not constant after treatment for some of the animals. In experiments with bleo, tirapazamine or VP16 alone, or with combined tirapazamine and bleo or combined tirapazamine and VP16, a marked decrease in tumour growth was noted at day 30 (Figure 2). The effect was present in one-third of the animals and in these cases the calculated volume doubling time did not reflect precisely tumour growth. The specific growth delay observed with the combination of tirapazamine and VP16 was only 0.5.

*Excision assay (Tables IV and V)*

For HRT18, the cell yield was significantly decreased with the combination of tirapazamine and bleo compared with the control group ($P=0.015$) (Table IV). However, an increase in cell yield was observed when tirapazamine was injected 3 h before bleo compared with simultaneous injection. The plating efficiency was significantly decreased with bleo alone ($P=0.001$), tirapazamine and bleo ($P=0.0001$) and tirapazamine 3h before bleo ($P=0.001$) compared with the control group. The combination of tirapazamine and bleomycine was more cytotoxic than tirapazamine alone ($P=0.04$), but not different from bleomycine alone ($P=0.06$). There was no difference between tirapazamine given 3 h or immediately before bleomycin. The lowest relative clonogenicity was observed with the combination of tirapazamine and bleo given at the same time (0.12). The effect of the combination of tirapazamine and c-DDP was comparable with tirapazamine alone, and did not differ from the control group.

For the Na11+ cell line, there was no significant modification of the cell yield with VP16 and tirapazamine alone or combined (Table IV). A significant decrease in plating efficiency was observed with tirapazamine alone ($P=0.002$), and tirapazamine and VP16 ($P=0.0001$). Tirapazamine was more effective than VP16 ($P=0.005$), and the combination of tirapazamine and VP16 was more effective than VP16 alone ($P=0.0001$). There was no difference between tirapazamine given 3 h or immediately before VP16. The lowest relative clonogenicity was observed with the combination of tirapazamine and VP16 given at the same time (0.21). With c-DDP, no differences were detected for the cell yield in treated animals with the controls (Table V). There was a significant decrease in plating efficiency when tirapazamine and c-DDP were given at the same time ($P=0.04$), compared with c-DDP or tirapazamine alone. This effect was not significant with a 3 h interval between the injections.

### Discussion

In patients, polarographic studies have shown large differences in oxygenation between normal tissues and tumours (Vaupel, 1990; Lartigau *et al.*, 1993). In tumours

**Figure 1** Tumour growth for xenografted rectal carcinoma (HRT18) after control (△-△), tirapazamine (■-■), bleo (- - -) or tirapazamine and bleo (□-□) injections. Pooled values for three different experiments (mean values and 95% confidence intervals are shown).

**Figure 2** Tumour growth for xenografted melanoma (Na11+) after tirapazamine and VP16 injections. Five individual tumours are presented.

**Table IV** Cell yield and plating efficiency after four daily injections of tirapazamine, bleo and VP16

| | Cell yield (mean × $10^6$, 95% CI) | Plating efficiency (mean, 95% CI) | Relative clonogenicity (s.d.) |
|---|---|---|---|
| *HRT18* | | | |
| Control (saline) | 3.13 (1.38–4.88) | 0.51 (0.39–0.62) | – |
| Tirapazamine (36)[a] | 2.41 (1.32–3.50) | 0.35 (0.20–0.49) | 0.53 (±0.28) |
| Bleo (25) | 1.76 (0.84–2.68) | **0.17*** (0.13–0.20) | 0.18 (±0.14) |
| Tirapazamine (36) + bleo (15) | **1.61*** (0.17–3.40) | **0.12*** (0.04–0.19) | 0.12 (±0.21) |
| Tirapazamine (36)- 3h–bleo (15) | 4.12 (1.88–6.36) | **0.09*** (0.04–0.14) | 0.24 (±0.11) |
| *Na 11+* | | | |
| Control (saline) | 0.38 (0.17–0.60) | 0.20 (0.14–0.26) | – |
| Tirapazamine (36) | 0.30 (0.21–0.38) | **0.09*** (0.06–0.12) | 0.35 (±0.37) |
| VP16 (20) | 0.31 (0.21–0.40) | 0.18 (0.13–0.23) | 0.73 (±0.40) |
| Tirapazamine (36) + VP16 (20) | 0.33 (0.22–0.43) | **0.05*** (0.03–0.07) | 0.21 (±0.18) |
| Tirapazamine (36)- 3h–VP16 (20) | 0.22 (0.12–0.32) | **0.09*** (0.05–0.12) | 0.26 (+0.18) |

[a]Figures in brackets represent daily dose in mg $kg^{-1}$. *Statistically significant difference compared with control.

**Table V** Mean cell yield and plating efficiency after one day injection of tirapazamine and cisplatinum for Na11+

| | *Cell yield (mean × $10^6$, 95% CI)* | *Plating efficiency (mean, 95% CI)* | *Relative clonogenicity (s.d.)* |
|---|---|---|---|
| Control (saline) | 0.72 (0.2–1.46) | 0.16 (0.05–0.49) | – |
| Tirapazamine (36)[a] | 0.79 (0.2–1.6) | 0.10 (0.03–0.38) | 0.71 (±0.37) |
| c-DDP (8) | 0.42 (0.3–0.55) | 0.05 (0.02–0.11) | 0.19 (±0.50) |
| Tirapazamine (36) +c-DDP (8) | 1.15 (0.6–2.24) | **0.01*** (0.003–0.03) | 0.10 (±0.21) |
| Tirapazamine (36)-3 h–c-DDP (8) | 0.37 (0.2–0.54) | 0.03 (0.01–0.11) | 0.10 (±0.33) |

[a]Figures in brackets represent daily dose in mg $kg^{-1}$. *Statistically significant difference compared with control.

$Po_2$ variations have been found from values superior to 20 mmHg, down to very low values (below 2 mmHg), with large variations between tumours (Gatenby *et al.*, 1988; Vaupel, 1990; Lartigau *et al.*, 1993; Höckel *et al.*, 1993). Very low $Po_2$ values can represent more than 25% of the recorded values in metastatic neck nodes (Lartigau *et al.*, 1993), but are very uncommon in normal tissues (Vaupel, 1990; Lartigau *et al.*, 1993, 1994). Thus, tumour hypoxia could represent a target for anti-cancer therapies, with the aim of being more specifically cytotoxic against hypoxic clonogenic tumour cells. Some anti-cancer agents, so-called 'bioreductive drugs', are activated in toxic metabolites only in the case of low $Po_2$. Their cytotoxicity in patients will depend on the drug concentration in the tumour, on the drug reduction as a function of the enzyme concentration and on the oxygen tension in the tumour (Bremmer, 1990; Koch, 1993; Brown, 1993; Workman and Stratford, 1993). One of the bioreductive drugs currently being tested in phase I in man (Doherty *et al.*, 1994) is tirapazamine (SR-4233). Tirapazamine has a lower hypoxic cytotoxic ratio in human cell lines (between 15 and 50) than in murine cell lines (Zeman *et al.*, 1986), and it has recently been shown that tirapazamine toxicity extends over a large range of oxygen concentrations (Costa *et al.*, 1989; Koch, 1993; Lartigau and Guichard, 1995).

To improve tumour control, the combination of radiotherapy and chemotherapy is currently strongly advocated (Schilsky, 1992; Rockwell, 1992). The bioreductive drugs could be associated to agents more cytotoxic in air than in hypoxia (bleo, procarbazine, vincristine), or to agents without any selective cytotoxicity according to tissue oxygenation (5-FU, c-DDP) (Teicher *et al.*, 1981). Before clinical implementation, such combinations have first to be tested in relevant experimental models.

Concerning mouse lethality, a 10% lethality with tirapazamine alone was obtained for a total injected dose of around 500 mg $m^{-2}$, corresponding to four successive daily injections of 0.20 mmol $kg^{-1}$ or 36 mg $kg^{-1}$. These results are similar to those obtained by others with rodent tumours (Zeman *et al.*, 1986; Minchinton *et al.*, 1992; Spiegel *et al.*, 1993). However, it must be emphasised that, in this study, the combination of tirapazamine and other chemotherapy agent always increased mice lethality. It was necessary to decrease the LD 10% for all the drugs in order to retain the LD 10% dose of tirapazamine. This was particularly true for 5-FU which produced high gastrointestinal toxicity (diarrhoea) with animal cachexia.

Concerning tumour growth delay, a marked effect was noted only with the combination of tirapazamine and bleo for the rectal adenocarcinoma, even with different injection times. This shows that two parameters are crucial: the drugs used and the tumour assessed. In our experiments, only the combination of a drug active against hypoxic cells (tirapazamine) and a drug active against aerobic cells (bleo) was efficient in a tumour model with a relatively low percentage of hypoxic cells (15–20%). Tirapazamine seems to have enhanced the activity of a drug (bleo) that was already very active on most of the cells present in the tumour. For the other drugs, in the two different tumour cell lines, the effect was not so pronounced. These results are similar to results obtained by other groups, which have consistently shown a limited effect of tirapazamine alone in tumour growth delay assays with murine or human cell lines (Minchinton *et al.*, 1992; SF Chen, personal communication). For a human colon cell line (HT29), tirapazamine or c-DDP had only marginal anti-tumour effect in an *in vivo* tumour growth delay assay (SF Chen, personal communication). For Na11+, a modification in tumour growth was noticed with delayed cytotoxicity. However, for this cell line the calculated volume doubling time and specific growth delay did not reflect precisely the biological effect of the drugs. The biological effect was in fact larger than that appreciated only with the calculation of the tumour doubling time. The late decrease in tumour volume, noticed at day 30 only, may have depended on the time necessary for this anoxic tumour to eliminate the dead cells.

For the excision assay, some effects on plating efficiency were noticed with the combination of drugs found to be the most active in the growth delay assay: tirapazamine and bleo for HRT18 and tirapazamine and VP16 for Na11+. However, the effect of these drugs were not as marked as those previously published by Holden or Dorie, who showed a very marked increase in toxicity when tirapazamine was injected 3 h before c-DDP (Holden *et al.*, 1992; Dorie and Brown, 1993). In particular, the timing of administration was not crucial in our experiments and the administration of tirapazamine 3 h before the other drug did not increase significantly tumour cell kill. We have no explanation for these differences. However, it must be emphasised that variations in experimental results can be found when using different tumour models, as for example between human and murine cell lines (Guichard, 1989). For the combination of tirapazamine and c-DDP a marked increase in plating efficiency was noted for HRT18, with the opposite effect for Na 11+. We have no explanation for such a result, but we feel that it should lead to more caution about using such a combination in patients.

In conclusion, tirapazamine was shown to be cytotoxic *in vivo* against clonogenic tumour cells. This efficacy may depend on the combination of tirapazamine with already active chemotherapy drugs on the tumour model studied (bleo on HRT18 and VP16 on Na11+). Some late effect on tumour growth was found in one of our xenografted tumours (Na11+), this not being precisely demonstrated by the calculation of the volume doubling time. Thus, the model used is very important to describe precisely the biological effect observed. Finally, the timing of drug administration and combination could be less important than previously thought (Dorie and Brown, 1993).

**Acknowledgements**
We wish to thank Mrs F Kokot for her secretarial assistance, Dr H Randrianarivello for his help, Mrs V Frascogna for her technical assistance and Mrs C Laudebat for the animal care. This work was supported by INSERM (U. 247), by grant IGR-CRC no. 91D8, by the 'Ligue Nationale contre le Cancer' (Comité de l'Essonne et Comité des Hauts-de-Seine), by the 'Association pour la Recherche contre le Cancer' and by Sterling Drug Inc.

### References

BREMMER JCM, STRATFORD IJ, BOWLER, J. AND ADAMS G. (1990). Bioreductive drugs and the selective induction of tumour hypoxia. *Br. J. Cancer*, **61**, 717–721.

BROWN JM. (1993). SR-4233 (tirapazamine): a new anticancer drug exploiting hypoxia in solid tumours. *Br. J. Cancer*, **67**, 1163–1170.

BROWN JM AND LEMMON MJ. (1991). Tumor hypoxia can be exploited to preferentially sensitize tumours to fractionated irradiation. *Int. J. Radiat. Oncol. Biol. Phys.*, **20**, 457–461.

COLEMAN CN. (1988). Hypoxia in tumours: a paradigm for the approach to biochemical and physiological heterogeneity. *J. Natl Cancer Inst.*, **80**, 310–316.

COSTA AK, BAKER MA, BROWN JM AND TRUDELL JR. (1989). *In vitro* hepatotoxicity of SR-4233 (3-amino-1,2,4-benzotriazine-1,4-dioxide) a hypoxic cytotoxin and potential antitumour agent. *Cancer Res.*, **49**, 925–929.

DOHERTY N, HANCOCK SL, KAYE S, COLEMAN CN, SHULMAN L, MARQUEZ C, MARISCAL C, RAMPLING R, SENAN S AND ROEMELING RV. (1994). Muscle cramping in phase I clinical trials of tirapazamine (SR-4233) with and without irradiation. *Int. J. Radiat. Oncol. Biol. Phys.*, **29**, 379–382.

DORIE MJ AND BROWN JM. (1993). Tumor-specific, schedule-dependent interaction between tirapazamine (SR-4233) and cisplatin. *Cancer Res.*, **53**, 4633–4636.

GATENBY RA, KESSLER HB, ROSENBLUM JS, COIA LR, MODOF SKY PJ, HARTZ WH AND BRODER GJ. (1988). Oxygen distribution in squamous cell carcinoma metastases and its relationship to outcome of radiation therapy. *Int. J. Radiat. Oncol. Biol. Phys.*, **10**, 831–838.

GUICHARD M. (1989). Comparison of the radiobiological properties of human tumour xenografts and rodent tumours. *Int. J. Radiat. Biol.*, **56**, 583–586.

GUICHARD M, GOSSE C AND MALAISE EP. (1977). Survival curve of a human melanoma in nude mice. *J. Natl Cancer Inst.*, **58**, 1665–1669.

GUICHARD M, DERTINGER H AND MALAISE EP. (1983). Radiosensitivity of four human tumor xenografts. Influence of hypoxia and cell-cell contact. *Radiat. Res.*, **95**, 602–609.

HÖCKEL M, VORNDRAN B, SCHLENGER K, BAUSSMANN E AND KNAPSTEIN PG. (1993). Tumor oxygenation: a new predictive parameter in locally advanced cancer of the uterine cervix. *Gynecol. Oncol.*, **51**, 141–149.

HOLDEN SA, TEICHER BA, ARA G, HERMAN TS AND COLEMAN CN. (1992). Enhancement of alkylating agents activity by SR-4233 on the FSaIIC murine fibrosarcoma. *J. Natl. Cancer Inst.*, **84**, 187–193.

KOCH CJ. (1993). Unusual oxygen concentration dependence of toxicity of SR-4233, a hypoxic cell toxin. *Cancer Res.*, **53**, 3992–3997.

LARTIGAU E, LE RIDANT AM, LAMBIN P, WEEGER P, MARTIN L, SIGAL R, LUSINCHI A, LUBOINSKI B, ESCHWEGE F AND GUICHARD M. (1993). Oxygenation of head and neck tumors. *Cancer*, **71**, 2319–2325.

LARTIGAU E, RANDRIANARIVELO H, MARTIN L, STERN S, THOMAS CD, GUICHARD M, WEEGER P, LE RIDANT A-M, LUBOINSKI B, NGUYEN T, ORTOLI J-C, GRANGE F, AVRIL M-F, LUSINCHI A, WIBAULT P, HAIE-MEDER C, GERBAULET A AND ESCHWEGE F. (1994). Oxygen tension measurements in human tumors: the Institut Gustave-Roussy experience. *Radiat. Oncol. Invest.*, **1**, 285–291.

LARTIGAU E AND GUICHARD M. (1995). Does tirapazamine (SR-4233) have any effect on the surviving fraction of 3 human cell lines at clinically relevant partial oxygen pressure? *Int. J. Radiat. Biol.*, **21**, 211–216.

MINCHINTON AL, LEMMON MJ, TRACY M, POLLART DJ, MARTINEZ AP AND BROWN JM. (1992). Second-generation 1,2,4-benzotriazine 1,4-di-N-oxide bioreductive anti-tumour agents: pharmacology and activity *in vitro* and *in vivo*. *Int. J. Radiat. Oncol. Biol. Phys.*, **22**, 701–705.

OVERGAARD J. (1991). Importance of tumour hypoxia in radiotherapy. A meta-analysis of controlled clinical trials. *Radiother. Oncol.*, **24**, S64.

ROCKWELL S. (1992). Use of hypoxia-directed drugs in the therapy of solid tumors. *Semin. Oncol.*, **19**, 29–40.

ROCKWELL S AND MOULDER JE. (1990). Hypoxic fractions of human tumors xenografted into mice: a review. *Int. J. Radiat. Oncol. Biol. Phys.*, **19**, 197–202.

SAKATA K, TAK KWOK T, MURPHY BJ, LADEROUTE KR, GORDON GR AND SUTHERLAND RM. (1991). Hypoxia-induced drug resistance: comparison to P-glycoprotein-associated drug resistance. *Br. J. Cancer*, **64**, 809–814.

SCHILSKY RL. (1992). Biochemical pharmacology of chemotherapeutic drugs used as radiations enhancers. *Semin. Oncol.*, **19**, 2–7.

SPIEGEL JF, SPEAR MA AND BROWN JM. (1993). Toxicology of daily administration to mice of the radiation potentiator SR 4233. *Radiother. Oncol.*, **26**, 79–81.

STEEL GG AND PECKHAM MJ. (1992). The therapeutic response of a variety of human tumour xenografts. In *Human Tumour Xenografts in Anticancer Drug Development*, Winograd B, Peckham MJ and Pinedo HM (eds) pp. 4–9. Springer: London.

TEICHER BA, LAZO JS AND SARTORELLI AC. (1981). Classification of antineoplastic agents by their selective toxicities towards oxygenated and hypoxic tumour cells. *Cancer Res.*, **41**, 73–81.

UCKCCCR. (1988). Guidelines for the Welfare of Animals in Experimental Neoplasia. *Br. J. Cancer*, **58**, 109–113.

VAUPEL PW. (1990). Oxygenation of human tumors. *Stralenther. Onkol.*, **166**, 377–386.

WEISSBERG JB, SON YH, PAPAC RJ, SASAKI C, FISCHER DB AND LAWRENCE R. (1989). Randomized clinical trial of mitomycin C as an adjunct to radiotherapy in head and neck cancer. *Int. J. Radiat. Oncol. Biol. Phys.*, **17**, 3–9.

WORKMAN P AND STRATFORD IJ. (1993). The experimental development of bioreductive drugs and their role in cancer therapy. *Cancer Met. Rev.*, **12**, 73–82.

ZEMAN EM, BROWN JM, LEMMON MJ, HIRST VK AND LEE WW. (1986). SR-4233: a new bioreductive agent with high selective toxicity for hypoxic mammalian cells. *Int. J. Radiat. Oncol. Biol. Phys.*, **12**, 1239–1242.

ZEMAN EM, HIRST VK, LEMMON MJ AND BROWN JM. (1988). Enhancement of radiation-induced tumour cell killing by the hypoxic cell toxin SR-4233. *Radiother. Oncol.*, **12**, 209–218.

ZEMAN EM, LEMMON MJ AND BROWN JM. (1990). Aerobic radiosensitization by SR-4233 *in vitro* and *in vivo*. *Int. J. Radiat. Oncol. Biol. Phys.*, **18**, 125–132.